# Life-Threatening Laryngeal Edema and Hyponatremia during Hysteroscopy

**DOI:** 10.1155/2011/140381

**Published:** 2011-03-29

**Authors:** Barbara Wegmüller, Kerstin Hug, Charlotte Meier Buenzli, Bernd Yuen, Marco Maggiorini, Alain Rudiger

**Affiliations:** ^1^Medical Intensive Care Unit, University Hospital Zurich, Raemistraße 100, 8091 Zurich, Switzerland; ^2^Department of Anesthesiology, Balgrist University Hospital, 8008 Zurich, Switzerland; ^3^Gynecology and Obstetrics, Canton Hospital Nidwalden, 6370 Stans, Switzerland; ^4^Anesthesiology and Intensive Care, Canton Hospital Nidwalden, 6370 Stans, Switzerland; ^5^Intensive Care Unit, Hospital Bülach, 8180 Bülach, Switzerland

## Abstract

We report on a 43-year-old patient undergoing a hysteroscopic myomectomy. After 80 minutes of operation, the patient developed laryngeal edema, requiring emergency tracheostomy. Hyponatremia (serum sodium 78 mmoL/L) indicated an irrigation fluid absorption. The patient developed shock, acute respiratory distress syndrome, acute renal failure, and diffuse intravascular coagulopathy. Resuscitation including continuous venovenous hemodiafiltration was required. Finally, the patient made a full clinical recovery. Hysteroscopy usually has low risks. However, absorption of the irrigation fluid can result in life-threatening fluid overload and electrolyte disturbances. Accurate fluid balancing and limiting the operation time may prevent these complications.

## 1. Introduction

Hysteroscopy is a routine procedure for diagnosis and treatment of abnormal uterine bleeding and infertility assessment [1, 2]. Jansen et al. described an overall risk for hysteroscopy of 0.28% and a risk for hysteroscopic myomectomy of 0.75% [3]. However, it can result in potentially severe and life-threatening complications as reported in this case.

## 2. Case Description

A 43-year-old woman with intracavitary myomas underwent an elective hysteroscopy because of excessive uterine bleeding. She had hysteroscopic and laparotomic enucleation of myomas two and twelve years ago, respectively. A hysterectomy was recommended, but the patient refused.

**Figure 1:**
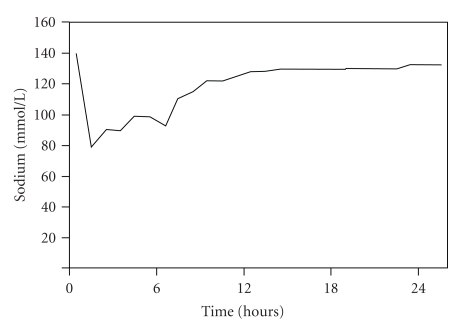
Postoperative correction of plasma sodium levels.

The preoperative clinical assessment revealed a healthy woman with American Society of Anesthesiology (ASA) physical status I. Preoperative laboratory testing including red cell and platelet counts, coagulation parameters, electrolytes, and renal function tests were normal (Table 1). Lumbar spinal anesthesia (L3/4) with 10 mg hyperbaric bupivacain 0.5% and supplemental 70 *μ*g clonidine was performed. Prior to the spinal puncture, the patient received an iv bolus of 0.1 mg fentanyl. A propofol-infusion (15–20 mg/h) was started when the anesthesized dermatome had reached T5. In addition, the patient received cefuroxim 1.5 g iv preoperatively. Standard monitoring including ECG, pulse oxymetry, noninvasive blood pressure, and urinary output measurements was installed. For optimal surgical visual field, the patient was placed in lithotomy position. The dilatation medium was a solution with 2.7% sorbitol and 0.54% mannitol, resulting in an osmolarity of 178 mosm/L (Purisole SM, Fresenius Kabi Germany). A hysteroscope (Storz, Germany) was used with regulation of inflow with a cuff at 100 mmHg. After 80 minutes of operation, a body temperature of 34°C and a fall of the oxygen saturation from 98% to <70% were recorded. Clinically, an acute pulmonary edema and swelling of the neck were apparent. The surgeon was informed and emergency intubation was indicated. An induction dose of propofol 100 mg, fentanyl 0.1 mg, and rocuronium 40 mg was administered. Because of massive laryngeal swelling, oral intubation failed. The vocal cords were not visible even with fiberoptic guidance. Finally, the placement of a laryngeal mask (LMA size 4 Laubscher, Switzerland) was successful, and an emergency tracheostomy was performed. By that time, 76 liters of irrigant fluid were used, but an exact irrigation fluid balance was not available. Urinary output was 1700 mL, and blood loss was only minimal. By then, she had received 1600 mL Ringer-Lactat (B. Braun, Switzerland) iv. The patient became hypotensive and required noradrenaline. Arterial blood analysis revealed an acute hyponatremia (78 mmoL/L) and an acidosis with a pH of 6.91 (Table 1, Figure 1). Treatment with iv furosemide, hypertonic NaCl, and bicarbonate 8.4% was initiated.

**Figure 2:**
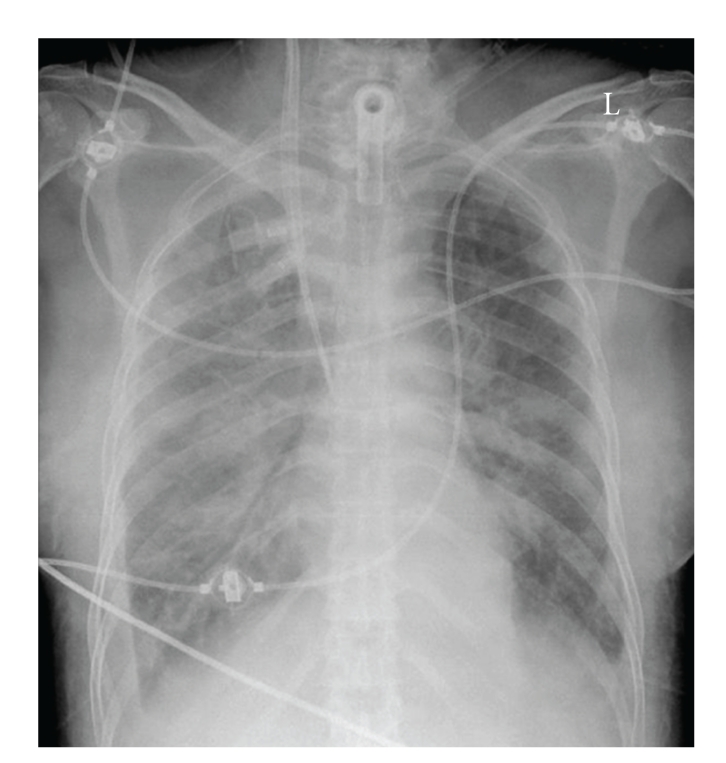
The chest X-ray on ICU admission reveals pulmonary edema.

For further treatment, the patient was referred to the intensive care unit of a tertiary medical center. On admission, the patient required high doses of catecholamines (noradrenaline up to 100 *μ*g/min, pitressin up to 0.02 U/min, and dobutamine up to 600 *μ*g/min). Her peripheral oxygen saturation was 81% with FiO_2_ 1.0, and the chest X-ray on admission revealed a pulmonary edema (Figure 2). The arterial blood gas analyses revealed the following values: pH 6.9, pCO_2_ 7.6 kPa, pO_2_ 7.8 kPa, bicarbonate 14.2 mmoL/L, base excess −17 mmoL/L, sodium 98 mmoL/L, chloride 77 mmoL/L, and lactate 5.3 mmoL/L. The osmolarity was normal, but the osmogap was 65 (Norm <5–10 mosmol/kg). Inhaled nitric oxide (5 ppm) was added, and oxygenation improved. Because sepsis was considered, piperacillin/tazobactam iv and low-dose corticosteroids were added. There was acute bleeding from the tracheostoma, epistaxis after insertion of a gastric tube, and uterine bleeding through an inflated Foley catheter placed intraoperatively. The administration of 8 packs of erythrocyte, 14 packs of fresh frozen plasma and one pack of platelets, 6 g of fibrinogen, 10 g of calcium, 5 g of aminocaproic acid, and 1000 *μ*g misoprostolum were necessary to stop the bleeding. In order to correct the acidosis and to remove the absorbed irrigation fluid, a continuous venovenous hemodiafiltration was started (dose 40 mL/kg bodyweight, anticoagulation with citrate, and blood flow 120 mL/min). The patient was sedated with midazolam. Her corneal reflexes were initially absent. An electroencephalography showed no signs of an encephalopathy although the blood ammonia level was 150 *μ*mol/l. Hypothermia of 33.2°C was still present.

Fortunately, the clinical course was favorable: the disseminated intravascular coagulopathy remained under control without any signs of further bleeding. Hyponatremia was corrected within 36 hours (Figure 1). A computed tomography scan of the head showed no cerebral edema, bleeding, or ischemia. After sedation stop, the patient woke up without any apparent neurological deficit. By day 7, the patient was weaned from the ventilator. However, due to the swelling of the neck, the tracheostomy canula was left *in situ* as long as the patient was in the ICU. Renal function recovered, and acid-base metabolism was reestablished with continuous haemodiafiltration, which was running for a total of 5 days. Vasopressor support with noradrenaline was required as long as the patient was on renal replacement therapy. The patient was discharged from the ICU on day 10 and from the hospital on day 30. One year later, she was well without any signs of long-term complications.

## 3. Discussion

During hysteroscopy, an irrigation fluid is necessary for dilatation and visualization the operating field. Fluid absorption appears to be more common during hysteroscopy than during transurethral resection of the prostate [4–6], the average amount being around 400–600 mL. The principle mechanism of fluid absorption is direct absorption of the irrigating fluid into opened vessels during hysteroscopic resection of the myoma. The driving force is the intrauterine fluid pressure, which can be higher than the hydrostatic venous and arterial pressure. The intrauterine pressure can be influenced by the position of the fluid bag in relation to the patient and adjustments of the hysteroscope. The amount of fluid absorption is also influenced by the wound size and the resection time [1, 7, 8]. Hence, there is a correlation between the size of the resected myoma and the amount of fluid absorption [7]. Our patient had a preoperative therapy with goserelinum in order to reduce the volume and vascularisation of the myoma. Nevertheless, the operation time exceeded the initially scheduled 60 minutes. Our case report supports the recommendation that the operation time for hysteroscopy should not be more than one hour. However, relevant fluid absorption can occur after only 15 minutes [9].

A preventive measure to recognize fluid absorption is an exact determination of the fluid balance by measuring the amount of fluid instilled into the uterus and the amount of fluid returned to the collection containers. It is recommended that the operative procedure is terminated when the fluid deficit reaches 1000 to 2000 mL [10]. At the time of symptoms, a total of 76 liters of irrigant fluids were used in our patient, which clearly exceeds the usually used 4–7 liters. Using the formula proposed by Serocki et al., we calculated a fluid overload of 11 liters [11]. This hypervolemia lead to the laryngeal swelling. The systemic inflammatory response syndrome with multiple organ failure might have resulted from hypoxemia, hypothermia, and fluid overload. To avoid fluid overload, restrictions of iv fluid and the use of diuretics are recommended during a longer procedure [7]. Serum electrolytes should be measured repeatedly, when more than 1000 mL are absorbed [7]. 

When hyponatremia develops within 24 hours, a rapid correction is indicated to prevent cerebral edema [12]. In our patient, plasma sodium was corrected within 36 hours, and there was no evidence of brain swelling. Premenopausal women with hyponatremia-induced postoperative encephalopathy have a higher morbidity and mortality and more frequently residual neurological damage than men or postmenopausal women [13]. However, in chronic hyponatremia, the correction of the plasma sodium concentration has to be less than 8 mmoL in 24 h in order to prevent pontine myelinolysis [14]. 

Some specific symptoms depend on the solution used for hysteroscopy. For monopolar resectoscopes, an electrolyte-free, hypotonic, and nonconductive solution is used (glycine 1.5% solution, sorbitol 2.7% with mannitol 0.54% mixture solution, or a mannitol 5% solution). With glycine, there is a dose-dependent increase of symptoms such as visual disturbance (glycine is an inhibiting neurotransmitter) or excess of ammonia [15–17]. Sorbitol is metabolised to fructose and glucose. After metabolism, sorbitol and glycine leave free water behind. As they both dilute in the extra- and intracellular compartments, cellular edema and dilutional hyponatremia are the consequences. Mannitol is inert, distributes only in the extracellular space, and is excreted unchanged by the kidney. Mannitol 3% has the largest sorbitol-mannitol mixture a medium and glycine 1.5% the smallest plasma dilutional effect [18, 19]. The osmotic-diuretic effects of the substances lead to even more sodium loss and thereby worsen hyponatremia. With a monopolar resectoscope, it seems to be safest to use mannitol or sorbitol/mannitol mixtures because of fewer neurological and cardiovascular compromises [8]. With a bipolar resectoscope it is possible to use isotonic electrolyte solutions. However, the risk of excessive fluid absorption is still present. 

This case emphasizes the importance of preventive measures in order to avoid a fluid absorption syndrome during hysteroscopy. First, close clinical observation and good communication between the anesthetist, patient, surgeon, and nurses during an endoscopic procedure are mandatory. This is facilitated with a regional anesthetic procedure. Second, an exact determination of the fluid balance is necessary. It is recommended that the procedure is terminated when the fluid absorption reaches 2000 mL [10]. Besides, restriction of iv fluids and the use of diuretics during a longer procedure are recommended [7]. Third, the resection time should not exceed 60 minutes [20] although the operation time seems to be less important than the total amounts of fluids used [8].

##  Financial Support

Intramural departmental sources.

##  Conflict of Interests 

The authors have no conflict of interests.

## Figures and Tables

**Table 1:** Laboratory parameters.

Laboratory parameters	Baseline	During surgery	On ICU admission	After 24 h	ICU discharge
Sodium—mmoL/L (Norm 136–145)	139	78	98	132	137
Chloride—mmoL/L (Norm 86–110)	—	—	77	104	110
Osmolality—mmoL/kg (Norm 280–300)	—	—	284	286	291
pH (Norm 7.37–7.47)	—	6.91	6.90	7.35	7.45
Base excess—mmoL/L (Norm ±2)	—	−16.7	−17.0	−2.0	1.1
HCO_3_^−^—mmoL/L (Norm 23–27)	—	16.5	14.2	22.8	24.2
Platelet count—1'000/*μ*l (Norm 143–400)	169	160	207	28	171
D-dimer—*μ*g/mL (Norm < 0.50)	—	8.59	6.36	21.36	2.40
